# Blood volume and chronic kidney disease in heart failure – Can volume expansion help balance the Cardio‐Renal Axis for better clinical outcomes?

**DOI:** 10.14814/phy2.15526

**Published:** 2022-12-02

**Authors:** Wayne L. Miller, Marat Fudim, Brian P. Mullan

**Affiliations:** ^1^ Department of Cardiovascular Medicine Mayo Clinic Rochester Minnesota USA; ^2^ Division of Cardiology Duke University Medical Center Durham North Carolina USA; ^3^ Division of Diagnostic Radiology and Nuclear Medicine Mayo Clinic Rochester Minnesota USA

**Keywords:** chronic heart failure, chronic kidney disease, outcomes, total blood volume, volume expansion

## Abstract

Intravascular volume is largely regulated by the kidneys but how differences in intravascular volume profiles interact with chronic kidney disease (CKD) to influence outcomes in chronic heart failure (HF) has not been explored. Our hypothesis was that a greater degree of volume expansion (VE) would moderate the impact of CKD on HF‐related clinical outcomes. Quantitative blood volume (BV) data were available in 137 patients at the time of hospital discharge using a nuclear medicine radiolabeled albumin indicator‐dilution technique. The study patients were stratified by the cohort median glomerular filtration rate (GFR, 44 ml/min/1.73 m^2^). An a priori cut‐point of ≥+25% above normal BV was then used to further stratify the two GFR subgroups and prospectively analyzed for 1‐year HF‐related mortality or 1st re‐hospitalization. Persistent BV expansions ≥+25% were present in 51% of the cohort. In the subgroup with GFR above the median (*N* = 68) greater or lesser BV expansion from +25% did not differentiate outcomes. However, in the subgroup with GFR below the median (*N* = 69), BV expansion‐stratified risk (log‐rank *p* = 0.022) with <+25% VE associated with poorer outcomes, while VE ≥ + 25% was associated with lower risk and comparable to GFR above the median. In patients with chronic HF, significant intravascular VE and CKD are common co‐existing conditions. The presence of larger VE, however, appears to be a factor mitigating the impact of declining renal function on clinical outcomes, and as an element of volume pathophysiology warrants further study.

## Graphical abstract

In patients with chronic heart failure (HF), intravascular volume expansion, and chronic kidney disease (CKD) are common co‐existing conditions. A large expansion in blood volume, however, functions to mitigate the impact of declining renal function on clinical outcomes. The beneficial buffering effect of volume expansion and the detrimental consequences of inadequate volume status in the clinical context of CKD and HF are elements of volume pathophysiology that warrant further study.

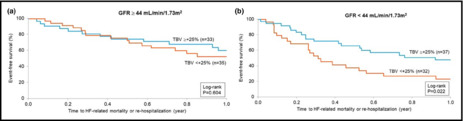

## 1 INTRODUCTION

Volume overload and symptomatic clinical congestion in association with chronic kidney disease (CKD) have been linked to poor prognosis in patients with chronic heart failure (HF). (Damman et al., 2010; Lucas et al., 2000; Testani et al., 2010; Kociol et al., 2013; Loffler et al., 2015; Damman et al., 2014; Bansal et al., 2019) While it is recognized that intravascular volume is largely regulated by the kidneys, the extent, and composition of volume expansion (VE) in relation to renal function on outcomes has not been explored. We and others have reported marked heterogeneity in the extent of blood volume (BV) expansion in patients with HF even where similar clinical presentations and interventions were identified. This variability includes not only heterogeneity in plasma volume (PV) but also in red blood cell (RBC) mass (Ahlgrim et al., 2020; Androne et al., 2004; Miller & Mullan, 2014; Miller & Mullan, 2015) and, importantly, potentially contrary to clinical expectations greater VE was associated with better HF‐related survival and fewer re‐hospitalizations (Miller et al., 2021). Here, we sought to investigate the potential mitigating impact of this variability in BV expansion on the severity of CKD and the related impact on outcomes in patients with chronic HF. Our working hypothesis was that greater intravascular VE at the time of hospital discharge would be compensatory by mitigating the impact of CKD [defined by glomerular filtration rate (GFR)] and be associated with better clinical HF outcomes, while lesser VE would not spare the negative effects of CKD on HF outcomes.

## 2 METHODS

Patients with chronic HF admitted to hospital for clinically determined symptomatic volume overload, had undergone intensive diuretic therapy and quantitative BV measurement (Daxor Corp. BVA‐100, NY, NY) at the time of hospital discharge were prospectively followed. All patients were receiving standard oral HF medical therapy including beta‐blockers, angiotensin‐converting enzyme inhibitors or angiotensin receptor blockers, mineralocorticoid receptor antagonists, and oral diuretics at the time of discharge and were determined by the primary care team to be clinically decongested and ready for dismissal from hospital. Patient inclusion criteria were: (a) age > 18 years, (b) patients identified clinically with volume overload at the time of hospital admission with New York Heart Association functional Class III‐IVa status, (c) ischemic or non‐ischemic dilated cardiomyopathy HF etiology, (d) left ventricular ejection fraction measured within 6 months prior to index hospitalization. Exclusion criteria: (a) CKD requiring hemodialysis, (b) known renal artery stenosis, (c) females who were pregnant or of child‐bearing potential, (d) non‐treatable allergy to iodine. Patients requiring intensive care unit therapy such as intravenous inotropes or vasodilators were not included in this study. The study was approved by the Mayo Foundation Institutional Review Board.

Quantitative BV analyses using the indicator‐dilution principle were undertaken in the Mayo Clinic Nuclear Medicine Laboratory using a standardized, computer‐based, and clinically available method to administer low‐dose iodinated (I‐131) labeled albumin intravenously. The specifics of the radiolabeled albumin indicator‐dilution technique which requires about 50 min to complete have been previously reported. (Feldschuh, 1990; Katz, 2007; Miller & Mullan, 2014; Miller & Mullan, 2015) For this analysis, lesser BV expansion was defined pre hoc as measured volumes < +25% of the expected normal volume and greater volume expansion as ≥+25% of normal volume. Red blood cell mass (in liters) was calculated using mean body hematocrit (MBHct) corrected from peripheral venous hematocrit and adjusted for trapped plasma to derive the measure of RBC mass [(PV/1‐MBHct) x MBHct = RBC mass]. The technique has been validated against double‐labeled chromium‐tagged RBCs and I‐125 albumin with comparisons within ±1%. (Dworkin et al., 2007; Fairbanks et al., 1996) Intravascular volume status is reported as absolute values (in liters) and as a percent deficit (−) or excess (+) relative to expected normal volumes based upon measurements in a broad population of healthy individuals of diverse body composition adjusted for weight, sex, and age. (Feldschuh & Enson, 1977; Feldschuh & Katz, 2007) The cut‐point used to define large VE (BV ≥ + 25% of normal expected volume) was established a priori based upon prior reported data. (Feldschuh & Enson, 1977; Feldschuh & Katz, 2007).

Data are presented as mean ± SD or median with 25th and 75th interquartile range (IQR) for continuous variables and categorical variables are reported as frequency (percentage) or number in the category. To assess differences, volume variables were compared using Wilcoxon sign‐rank test for paired analyses, and percentage differences by chi‐squared analysis. Median values were compared using the Mann–Whitney test. One‐way ANOVA was used to test for significant differences between groups with *p* < 0.05 being considered statistically significant. Adjustments for multiple comparisons were not done, but p‐values are provided. Survival was estimated using the Kaplan–Meier (K–M) log‐rank analysis to test for differences in outcomes for the composite endpoint of HF‐related mortality or 1st HF‐related re‐hospitalization among groups. Patient survival or HF‐related mortality was confirmed using the Mayo Clinic, Rochester, electronic medical records system. First post‐index HF‐related re‐hospitalization was identified by manual surveillance of electronic medical records and ICD‐9‐CM codes 425 and 428 and ICD‐10‐CM 150 codes. One‐year follow‐up was complete to the date of study censor in all surviving and non‐re‐hospitalized patients. No deaths occurred during the index hospitalization. Renal function was expressed as the GFR, ml/min/1.73 m^2^, using the Modification of Diet in Renal Disease equation. (Levey et al., 2003) Statistical analyses were performed using SAS, version 9, statistical software (SAS Institute, Cary, North Carolina) and JMP 8 with *p*‐values <0.05 considered statistically significant.

## 3 RESULTS

**FIGURE 1:**
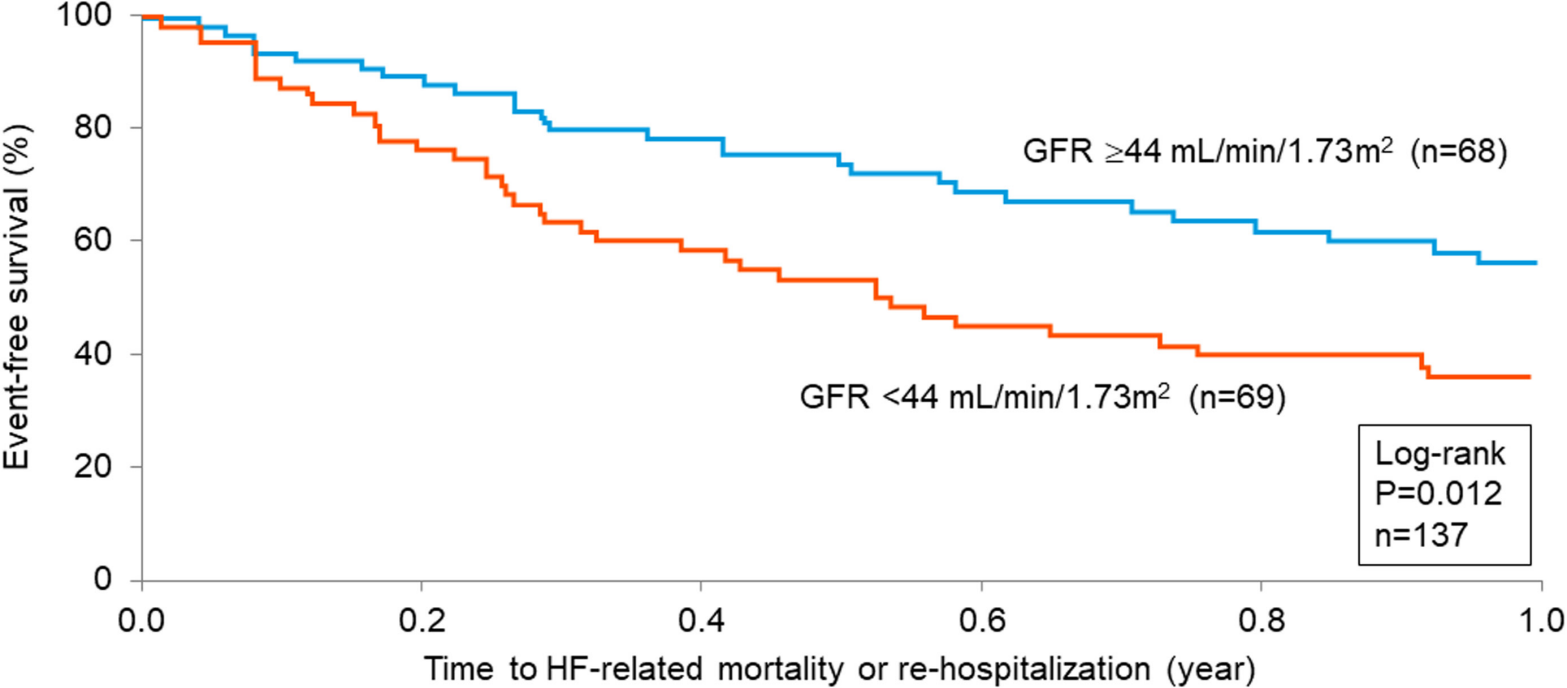
K–M estimates of event‐free survival in patients with chronic HF stratified by cohort median GFR (44 ml/min/1.73 m^2^).

Blood volume and clinical data were available on 137 patients who were prospectively followed post‐hospitalization over 1 year for the composite endpoint. The patient cohort was dichotomized based upon cohort median GFR of 44 ml/min/1.73 m^2^. Figure 1 shows the K–M survival estimates for the composite end point with significantly poorer outcomes with GFR below the median (log‐rank *p* = 0.012). Table 1 shows the clinical and demographic features of these two GFR subgroups. As expected, the median subgroup GFRs differed. Patients with lower GFR were older, lower hemoglobin concentrations, higher levels of creatinine, BUN and NT‐proBNP but were otherwise similar in clinical characteristics and features to the GFR subgroup above the median.

**TABLE 1:** Clinical and demographic characteristics stratified by glomerular filtration rate (cohort median) at hospital discharge

Variable	GFR ≥44 ml/min/1.73 m^2^ (*N* = 68)	GFR <44 ml/min/1.73 m^2^ (*N* = 69)	Intergroup comparison
eGFR, mL/min/1.73 m^2^, median	59 (51, 77)	27 (11, 36)	*p* < 0.001
Age, years	65 ± 15	71 ± 11	*p* = 0.011
Sex, M/F	48/20	50/19	*p* = 0.796
Body mass index, kg/m^2^	33 ± 8	32 ± 8	*p* = 0.514
Systolic blood pressure, mm Hg	113 ± 16	114 ± 16	*p* = 0.743
LVEF, %	36 ± 18	34 ± 16	*p* = 0.564
Length of hospital stay, days	5.3 ± 1.9	5.5 ± 1.8	*p* = 0.570
Diabetes, %	46%	48%	*p* = 0.815
Hypertension, %	72%	65%	*p* = 0.379
Coronary artery disease, %	53%	58%	*p* = 0.557
Atrial fibrillation, %	37%	51%	*p* = 0.101
Sleep apnea, %	60%	58%	*p* = 0.813
Hemoglobin, g/dl	12.5 ± 2.3	11.2 ± 1.9	*p* = 0.001
Hematocrit, %	39 ± 7	37 ± 6	*p* = 0.075
Serum creatinine, mg/dl	1.3 ± 0.4	2.2 ± 0.8	*p* < 0.001
BUN, mg/dl	29 ± 13	53 ± 21	*p* < 0.001
Sodium, mEq/L	139 ± 4.0	139 ± 3.8	*p* = 1.00
Potassium, mEq/L	4.2 ± 0.4	4.1 ± 0.4	*p* = 0.268
Plasma glucose, mg/dl	121 ± 40	122 ± 30	*p* = 0.869
NT‐proBNP, pg/ml	3842 (1718, 7626)	7545 (3981, 17,504)	*p* < 0.001

*Note*: Data expressed as Mean ± SD, percent of category, median with 25th, 75th IQR.

Abbreviations: GFR, glomerular filtration rate; IQR, interquartile range; LVEF, left ventricular ejection fraction.

Table 2 shows total BV data which when partitioned by median GFR did not differ between subgroups. Total BV when stratified by the cut‐point of +25% above normal expected volume identified 70/137 (51%) patients with BV ≥ + 25%. Those with BV < +25% expanded (*N* = 67/137) had either a normal BV (38/67, 57%) or mild to moderate expansion (29/67, 43%). Patients with BV expansion ≥ + 25% were more likely to have larger RBC mass on average regardless of GFR subgroup; however, in patients with BV expansion <+25%, GFR below the median was associated with low RBC mass (more anemia).

**TABLE 2:** Blood volume (BV) and red blood cell mass (RBC mass) stratified by cohort median glomerular filtration rate (GFR)

Variable	GFR ≥44 ml/min/1.73 m^2^ (*N* = 68)	GFR <44 ml/min/1.73 m^2^ (*N* = 69)	Intergroup comparison	
Blood volume, liters range	6.8 ± 1.7 3.2 to 8.9	6.6 ± 1.5 3.4 to 9.4	*p* = 0.466	
% Excess (+)/deficit (−) of normal range	+24 ± 21% −12% to +77%	+28 ± 24% −18% to +80%	*p* = 0.301	
BV ≥ + 25% of normal	N = 33 7.8 ± 1.5 L +41 ± 15%	*N* = 37 7.5 ± 1.1 L +46 ± 14%	*p* = 0.339	
BV < +25% of normal	*N* = 35 5.8 ± 1.2 L +8 ± 10%	*N* = 32 5.6 ± 1.4 L +6 ± 11%	*p* = 0.531	
	*p* < 0.001	*p* < 0.001		
RBC mass, liters range	2.4 ± 0.8 1.1 to 5.1	2.2 ± 0.7 1.2 to 4.4	*p* = 0.122	
% Excess (+)/deficit (−) of normal range	+12 ± 27% −34 to +107%	+4 ± 28% −40 to +89%	*p* = 0.091	
RBC mass when BV≥ + 25% above normal	*N* = 33 2.7 ± 0.8 L +22 ± 32%	*N* = 37 2.5 ± 0.6 L +22 ± 24%	*p* = 0.234	
RBC mass when BV < +25% of normal	*N* = 35 2.1 ± 0.6 L +3 ± 17%	*N* = 32 1.7 ± 0.5 L −18 ± 14%	*p* = 0.004	
	*p* = 0.001	*p* < 0.001		
RBC mass phenotype distribution, %	BV≥ + 25%	BV < +25%	BV≥ + 25%	BV < +25%
True anemia	24%	23%	3%	66%
Normal RBC mass	7%	26%	29%	31%
RBC mass polycythemia	69%	51%	68%	3%

*Note*: Data expressed as Mean ± SD, percent of category, median with 25th, 75th IQR.

Abbreviations: BV, blood volume; GFR, glomerular filtration rate; IQR, interquartile range; RBC, red blood cell.

When stratified by BV above and below +25%, absolute BVs were similar between GFR subgroups (Table 2), however, absolute BVs differed above and below +25% when assessed by within GFR subgroup. Additionally, Table 2 demonstrates similar data for RBC mass between GFR subgroups. There was a trend, however, for less deviation of the RBC mass from normal (+4% vs. +12%, *p* = 0.091) to be associated with GFR below the cohort median. Also, the lower GFRs where BV expansion was <+25% demonstrated significantly reduced RBC mass with a large component of true anemia (deficit in RBC mass relative to normal).

Table 2 also shows the frequency distribution of RBC mass profile identified in this patient cohort. In HF patients with GFR above the cohort median, RBC polycythemia (RBC mass excess) was the predominant feature (41/68, 60%) regardless of the extent of BV expansion. True anemia was present in only 16/68 (24%) of this GFR subgroup and was equally distributed between BV subgroups. In contrast, when GFR was below the median, RBC polycythemia was the most prominent profile only with BV expansion ≥ + 25% (68%, 25/37). Total BV was significantly greater in this subgroup reflecting both the RBC polycythemia as well as compensatory and pathologic PV expansion as contributors to the large VE. While marked heterogeneity in both PV and RBC mass profiles was evident, RBC mass excess was present in only 3% of the <+25% VE subgroup and true anemia was the predominant phenotype (66%, 21/32).

**FIGURE 2:**
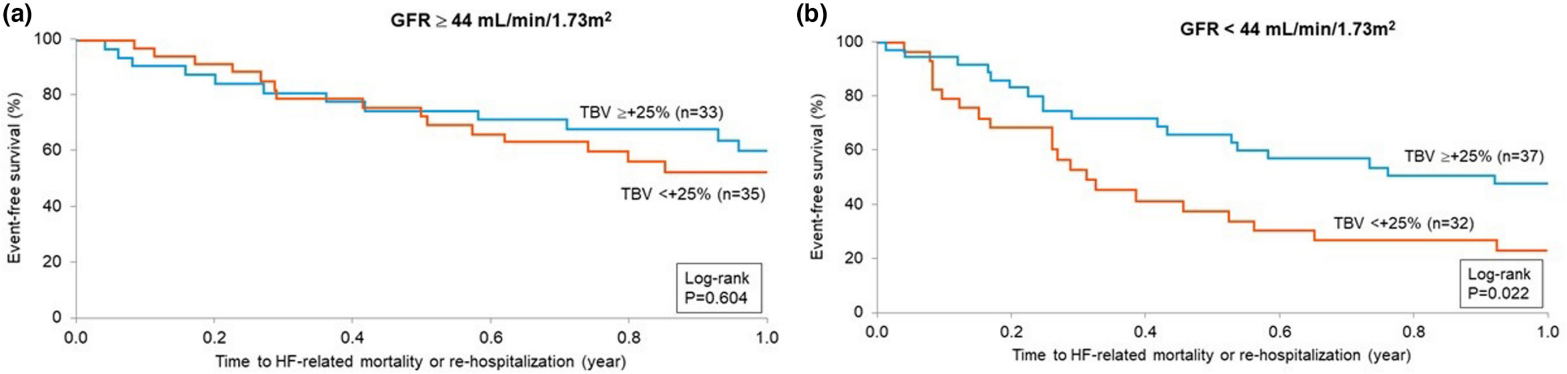
(a) K–M estimates of event‐free survival in chronic HF patients with GFR above cohort median (≥44 ml/min/1.73 m^2^) stratified by BV expansion above and below +25% of normal expected volume. (b) K–M estimates of event‐free survival in chronic HF patients with GFR below cohort median (<44 ml/min/1.73 m^2^) stratified by BV expansion above and below +25% of normal expected volume.

Figure 2 shows the survival relationship of BV expansion subgroups above and below +25% of normal in HF patients with GFR above the cohort median of 44 ml/min/1.73 m^2^. By K–M estimates there was no impact by the extent of BV expansion on outcome with an overall 1‐year survival of approximately 58% (log‐rank *p* = 0.604). In contrast, Figure 2B shows a significant separation of survival estimates in relation to the extent of BV expansion when GFR is below the cohort median. Patients with low GFR and less BV expansion experienced significantly poorer outcomes (approximately 25% survival at 1 year) relative to those patients with also a low GFR but an expanded BV (above +25%) where 1 year survival was approximately 50% (log‐rank *p* = 0.022).

## 4 DISCUSSION

The findings of this observational study provide first‐time data describing the interrelationship and impact of differences in intravascular volume profiles relative to the severity of CKD on HF‐related outcomes. In the overall cohort, VE was associated with better clinical outcomes; however, intravascular volume also appears to be closely related to the impact of CKD severity. The connection between the extent of intravascular VE and CKD has had limited discussion as an element in the pathophysiology of chronic HF. As a result, the variability in BV profiles has not been taken into account in risk assessment paradigms or viewed as a factor to modify in the management of HF patients with co‐existing CKD. In this study we addressed the interconnection of BV expansion with a standard clinical biomarker of renal function (GFR) on HF‐related outcomes including mortality. Our study findings support the observation that BV expansion from lesser to greater degrees interacts with the relative severity of renal dysfunction to modify clinical outcomes in patients with chronic HF. Greater BV expansion appears to buffer the risk of declining GFR. In contrast, lesser or inadequate VE did not spare the increased risk associated with more advanced CKD. Better renal function as reflected by a higher GFR (here above cohort median) in the setting of lesser BV expansion, however, appears to support relatively better outcomes. This is hypothesis generating based on these data and requires further study.

The variability in intravascular volume profiles is potentially a meaningful clinical metric influencing the impact of CKD on HF outcomes. The observation that intravascular VE in contrast to a normal volume or inadequate volume expansion mitigates the risk associated with CKD has implications for individualized patient management and risk stratification. These study findings support the concept that an optimal degree of BV expansion is needed to balance the risk of worsening renal function and act as a plausible mechanism to help stabilize kidney perfusion. The impact of intravascular VE may in part relate to the kidney's sensitivity to reduced perfusion pressure where a low renal medullary p0_2_ can be exacerbated by inadequate intravascular volume provoking greater renal hypoxia (Ding et al., 2017). In contrast, in the setting of VE with RBC polycythemia as presented in this cohort, O_2_ delivery and medullary pO_2_ would be expected to be favorable in countering the risk of worsening renal function. Therefore, VE acting as a compensatory mechanism may be a contributing factor to the observed better clinical outcomes.

The favorable impact of greater VE on CKD‐HF‐related outcomes appears to receive a substantial contribution from the presence of RBC mass excess (polycythemia) which would be a mechanism potentially supporting better tolerance of declining renal function. As suggested in previous analyses (Ahlgrim et al., 2020; Miller & Mullan, 2015) RBC mass excess is likely a compensatory response to tissue hypoxia, particularly the renal hypoxemia of HF‐related CKD, secondary to impaired cardiac output and compromised organ perfusion. Further investigation, however, is warranted to better define the roles of increased RBC mass, PV expansion, and associated mechanisms of erythropoiesis and potential blood viscosity changes in chronic HF. Also, as shown by these data is the impact of a deficit in RBC mass (true anemia) in patients with poorer renal function and less VE – 66% of patients with GFR below the median and BV expansion <+25% demonstrated true anemia which would be expected to be a contributor to the observed poorer outcomes in this subgroup. The quantitation of total BV to include assessment of RBC mass as a clinically available tool can thus be useful in identifying specific BV phenotypes and guide more targeted therapy in HF patients with concomitant CKD.

Additionally, relevant to HF clinical practice is the importance of recognizing the significance of volume‐renal interactions and that these 2 systems do not likely interact in the same manner across all stages of HF progression. Differences in how the extent of VE and renal function interact may point to the manner how mechanisms of compensation evolve over time, and that the contribution of VE to clinical stability will vary with progression of disease. The findings of this study are novel and hypothesis generating in providing a basis to further study the potential clinical significance of differing intravascular volume phenotypes relative to stages of CKD in patients at different stages of HF.

There are study limitations to consider. First, this is an observational single‐center study of prospectively collected data from a tertiary referral medical center with potential limitations of selection bias and generalizability of findings. Second, the generalization of the findings to all patients with HF and CKD (as indicated above) and suspected volume overload should not be done and requires additional study in appropriately defined HF and CKD patient cohorts. Third, the lack of serial BV and renal biomarker measurements over the time course of the study limit the ability to account for changes in BV status and renal function (cross‐over) which may have occurred and possibly influence risk assessment overtime. Fourth, while clinical features and characteristics were not different by patient cohorts, statistical adjustments for confounders were not done given the relatively low patient numbers in subgroups. Multi‐center studies would be of value to expand on and confirm these results.

In conclusion, the findings of this analysis support the observation that in patients with advanced HF significant intravascular VE is persistent and common even at hospital discharge when clinical compensation has been established. Importantly, a greater degree of intravascular VE, in contrast to an inadequate expansion or normal volume, appears to be compensatory by buffering the effects of declining GFR on outcomes. Therefore, the extent of BV expansion appears to be an important metric relative to the severity of CKD to impact HF‐related outcomes. Further, the observation that in patients no longer demonstrating clinical signs or symptoms of volume overload, better clinical outcomes are associated with an expansion in BV rather than a normal BV, even with concomitant CKD, suggests that the concept of what is an “optimal BV” in patients with chronic HF is still an issue to be defined.

## FUNDING INFORMATION

Unrestricted research grant from the Daxor Corporation, New York, NY, Feldschuh Foundation for Clinical Research, New York, NY, and Mayo Clinic Department of Cardiovascular Medicine research support.
